# Decoupling Ion–Dipole Interactions via Competitive Coordination for Durable Dendrite-Free Zinc Metal Batteries

**DOI:** 10.1007/s40820-026-02306-5

**Published:** 2026-08-10

**Authors:** Bixian Chen, Xiaomin Cheng, Xiang Li, Qinghua Guan, Hao Li, Yongzheng Zhang, Shuang Cheng, Huihua Li, Yunjian Liu, Jing Zhang, Hongzhen Lin, Jian Wang

**Affiliations:** 1 i-Lab & CAS Key Laboratory of Nanophotonic Materials and Devices, Suzhou Institute of Nano-Tech and Nano-Bionics, Chinese Academy of Sciences, Suzhou, 215123 People’s Republic of China; 2 Helmholtz Institute Ulm (HIU), 89081 Ulm, Germany; 3 Karlsruhe Institute of Technology (KIT), 76021 Karlsruhe, Germany; 4 School of Materials Science and Engineering, Jiangsu University, Zhenjiang, 212013 People’s Republic of China; 5 Anhui Provincial Key Laboratory of Green Carbon Chemistry, Fuyang Normal University, Fuyang, 236037 People’s Republic of China; 6 School of Textile & Clothing, Nantong University, Nantong, 226019 People’s Republic of China; 7 School of Materials Science and Engineering, Xi’an University of Technology, Xi’an, 710048 People’s Republic of China; 8 Guangdong Institute of Semiconductor Micro-Nano Manufacturing Technology, Guangdong, 518103 People’s Republic of China

**Keywords:** Aqueous zinc metal batteries, Reconstructed Helmholtz plane, Interface chemistry, Ion–dipole interaction, Zn dendrite inhibition

## Abstract

The aqueous zinc metal batteries (AZMBs) are famous for high-safety and high-energy-density, but limited by severe challenges around the Helmholtz plane layer such as the strong ion–dipole interactions between Zn^2+^ and H_2_O, resulting in slow desolvation processes and limited transport kinetics as well as corresponding higher barriers. To reconstruct the ion–dipole surroundings, the interface chemistry of employing a high permanent dipole moment of *L*-Carnosine (*L*-CN) has been proposed, weakening the interactions between Zn^2+^ and H_2_O to realize a crowded Zn^2+^-conductive structure, accelerating the desolvation kinetics. As revealed, the strong affinity between *L*-CN and Zn^2+^ enables the *L*-CN molecules to repulse H_2_O, reconfiguring the inner Helmholtz plane layer, thereby inhibiting the active water molecular to form hydrogen evolution reactions. Consequently, the Zn//Zn symmetric cells with Helmholtz plane modulation achieve a long lifespan up to 7000 h, a high Coulombic efficiency of 99.72%, and a high stabilization at high depth of discharge (85.4%). The assembled Zn//V_2_O_5−*x*_ full cell with optimal electrolyte delivers the capacity of 289 mAh g^−1^ after 500 cycles at 1 A g^−1^ under an N/P ratio of 3.98. Impressively, the large-scale pouch cell with optimal electrolyte stabilizes for 200 cycles, offering the bright future of reconstructing Helmholtz plane for achieving high-performance AZMBs.

**Supplementary Information:**

The online version contains supplementary material available at 10.1007/s40820-026-02306-5.

## Highlights

- A competitive permanent dipole *L*-Carnosine (*L*-CN) is introduced to interact with Zn^2+^ in the Zn^2+^–H_2_O solvation shell and form Zn^2+^(*L*-CN)_*y*_(H_2_O)_*x*_ structure, enhancing the desolvation kinetics of [Zn(H_2_O)_6_]^2+^ and diffusion kinetics of zinc ions/atoms.
- The *L*-CN molecule forms a crowded Zn^2+^-conductive structure and remodels the inner Helmholtz plane layer with abundant Zn^2+^ flux, enabling durable dendrite-free Zn deposition.
- The cells with optimal electrolyte stabilize for 7000 h, a high Coulombic efficiency of 99.72%, and even stabilize over 200 h under a high depth of discharge of 85.4%. The assembled Zn//V_2_O_5−*x*_ full cell delivers the capacity of 289 mAh g^−1^ after 500 cycles at 1 A g^−1^ under an N/P ratio of 3.98.

**Supplementary Information:**

The online version contains supplementary material available at 10.1007/s40820-026-02306-5.

## Graphical abstract

**Figure:**
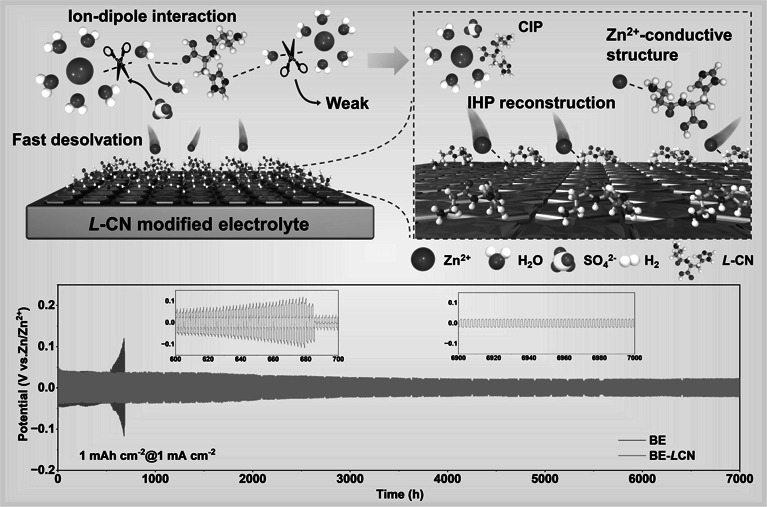

## Introduction

**Fig. 1:**
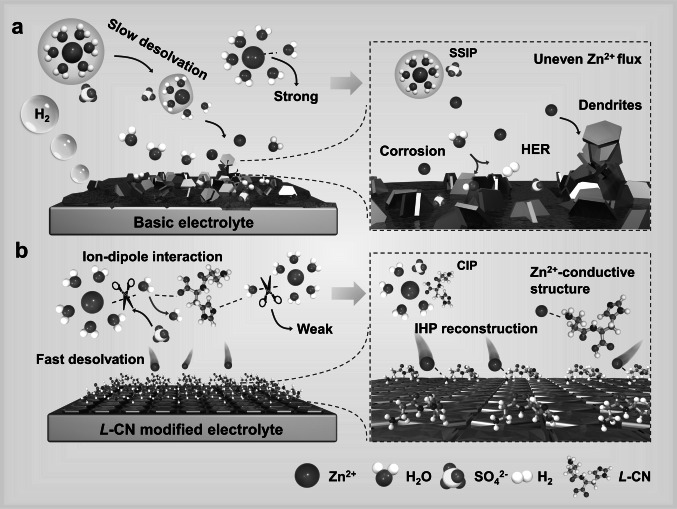
Schematic illustrations of Zn behaviors in **a** Basic electrolyte and **b** *L*-CN modified electrolyte, where the IHP is reconstructed

Aqueous zinc metal batteries (AZMBs) have emerged as promising next-generation energy storage systems owing to their inherent safety, low cost, and high volumetric energy density [1–6]. However, severe challenges such as low Coulombic efficiency (CE), insufficient Zn utilization, and dendrite formation hinder their commercialization progress [7–12]. These problems primarily stem from the strong ion–dipole interactions between Zn^2+^ and H_2_O in the Helmholtz plane [13–16], resulting in slow desolvation processes and limited transport kinetics (Fig. 1a). The mismatch with the electron and slow Zn^2+^ kinetics also disrupts the electric field distribution within the electric double layer (EDL), further leading to irregular Zn^2+^ deposition [17–20]. Meanwhile, partially dissociated Zn(H_2_O)_*x*_^2+^ tends to compete with Zn^2+^ plating for electrons, triggering the hydrogen evolution reaction (HER) and consuming the limited electrolyte [21–25].

To address the aforementioned challenges, traditional artificial interphase layers such as ZrO_2_ [26] and SnF_2_ [27, 28] or polymer-decorated inorganics [29, 30] were employed as physical barriers to average Zn^2+^ flux and isolate electric contact between H_2_O and Zn metal, effectively minimizing the effects of dendrite growth and HER. As known, in the typical aqueous electrolyte, water solvent molecules predominantly occupy the primary solvation, while anions are excluded from the Zn^2+^ solvation structure, forming the ion(Zn^2+^)–dipole(H_2_O) interactions and exhibiting a high desolvation energy barrier [5, 31–33]. Some electrolyte additives, such as polyethylene glycol (PEG) [34] and ZnCl_2_ [35], have been introduced and compete with H_2_O in the solvation shell, forming a water-lean solvation environment. However, these approaches suffer from sluggish ion transport kinetics and are at the expense of ionic conductivity [36]. Decoupling ion–dipole interactions can reduce the desolvation barrier for cations and enhance the solvation, thereby forming a mutated Helmholtz layer [37, 38]. Thus, to break down the Zn^2+^–H_2_O clusters, it is reasonable to weaken the solvation capability by competing with the interactions between Zn^2+^ and additive organics [39], accelerating the dissociation of Zn^2+^–H_2_O [40], even increasing the depth of discharge (DOD) state, due to the spatial hindrance effect, taming the ion–dipole interactions for Zn^2+^ transport seems to be of significance [41].

Herein, to reconstruct the ion–dipole behaviors in the Helmholtz layer, the interface chemistry of employing a high permanent dipole moment additive has been proposed from the electrolyte to the interface, weakening the interactions between Zn^2+^ and H_2_O to realize a crowded Zn^2+^-conductive structure and accelerating the desolvation kinetics. As representatively illustrated in Fig. 1b, the *L*-Carnosine (*L*-CN) molecule with various functional groups preferentially interacts with Zn^2+^ in the Zn^2+^–H_2_O solvation shell, which facilitates the desolvation process of Zn^2+^ by forming crowded Zn^2+^-conductive structure, remodeling the inner Helmholtz plane (IHP) layer with abundant Zn^2+^ flux [42]. As confirmed by multiple simulations, in-depth X-ray photoelectron spectroscopy (XPS), time-of-flight secondary-ion mass spectrometry (TOF–SIMS), and optical image assessments, the Zn anode with *L*-CN-based optimal electrolyte achieves a high average CE of 99.72% within 2000 cycles at 5 mA cm^−2^_,_ stabilizes over 7000 h at 1 mA cm^−2^, and is even operated at a high DOD of 85.4%. The optimized Zn//V_2_O_5−*x*_ full cell delivers high capacity of 289 mAh g^−1^ after 500 cycles at 1 A g^−1^ under a low N/P ratio of 3.98. Impressively, the large-scale pouch cell employed with *L*-CN additive operates stably for hundreds of cycles and powers the smart devices, highlighting the great potential for commercial applications of AZMB.

## Experimental Section

### Preparation of Electrolytes

The ZnSO_4_-based electrolyte was set as 2 mol L^−1^ (noted as BE). The *L*-CN additive was added into BE with the concentration ranging from 5 to 50 mmol L^−1^. Without any specific statement, the concentration in the modified electrolyte is set as 10 mmol L^−1^ (noted as BE-*L*CN). For the Zn//V_2_O_5−*x*_ cells test, the 3 M Zn(OTf)_2_ was selected as the pristine electrolyte and the addition of *L*-CN additive was set as 10 mmol L^−1^.

### Preparation of V_2_O_5−***x***_ Cathode

The fabrication of V_2_O_5−*x*_ cathode is similar according to our previous report [40]. Commercial V_2_O_5_ particles were treated at 400 °C for 2 h under a 5% H_2_/Ar atmosphere. Subsequently, the prepared V_2_O_5−*x*_, Super P, and polyvinylidene fluoride (PVDF) were mixed at a weight ratio of 7:2:1 in *N*-methyl-2-pyrrolidone (NMP) solvent to form a homogeneous slurry. The obtained slurry was uniformly coated onto carbon paper and dried at 60 °C for 6 h in a vacuum oven.

### Preparation of Polyaniline (PANI) Cathode

2.187 mL of aniline was added to 250 mL of 1 mol L^−1^ HCl and stirred for 30 min (Solution A). Then, 2 g carbon nanotubes (CNTs) and 5.472 g of ammonium persulfate ((NH_4_)_2_S_2_O_8_) were dissolved in 250 mL of 1 mol L^−1^ HCl and stirred at room temperature for 1 h (Solution B). Under an ice bath, Solution B was slowly added dropwise to Solution A under continuously stirring, and the reaction was allowed to proceed for 24 h. After completion, the product was washed for 3 times with water and dried at 40 °C to obtain PANI. For electrode preparation, PANI, Super P, and PVDF were mixed at an 8:1:1 mass ratio in NMP to form a homogeneous slurry. The obtained slurry was uniformly coated onto carbon paper and dried at 60 °C for 6 h in a vacuum oven.

### Assembly and Electrochemical Measurement of Cells

The Zn//Zn symmetric cells used Zn foils (10 mm in diameter) as electrodes. The half cells used copper or titanium (15 mm in diameter) and Zn foils as electrodes, respectively. The full cells employ V_2_O_5−*x*_ or PANI cathodes (10 mm in diameter) and Zn foils (12 mm in diameter) as electrodes. All test cells were assembled in CR2025 coin-type cells using a glass fiber GF/B separator (Whatman) and 150 µL of electrolyte. Before electrochemical testing, all assembled cells were rested at room temperature for 1 h to ensure complete electrolyte to wet the separator and electrode and to stabilize the electrode/electrolyte interface. The electrochemical cycling tests were recorded using a battery testing instrument (Land CT2001A). Tafel curves, chronoamperometry (CA), linear sweep voltammetry (LSV), electrochemical impedance spectroscopy (EIS), and cyclic voltammetry (CV) measurements were obtained on an electrochemical workstation (VMP-3, France).

The differential capacitance–potential curves were characterized by phase selective AC voltammetry on Zn||Cu cells at a scanning rate of 4 mV s^−1^ with amplitude (A) of 5 mV and frequency (f) of 6 Hz. The capacitance was calculated according to the equation: $C\, = \,\frac{1}{{2{\uppi }fZ_{{{\mathrm{im}}}} }}$, where *Z*_im_ = *Z*sinΦ, Φ = arctan($\frac{{i_{90} }}{{i_{0} }}$),$Z\, = \,\frac{A}{{\sqrt {i_{90}^{2} + i_{0}^{2} } }}$, and *i*_90_ and *i*_0_ are the currents at the phase angles of 0° and 90°, respectively. Tafel curves were obtained using Zn//Zn symmetric cells with a voltage window from 0.2 to − 0.2 V at a scan rate of 1 mV s^−1^. CA curves were measured using Zn//Zn symmetric cells under a bias of 150 mV for 300 s. LSV curves were measured using a standard three-electrode configuration with Ag/AgCl reference electrode with a voltage window from 2.5 to − 2 V at a scan rate of 1 mV s^−1^. EIS curves were measured in a frequency range of 200 kHz–0.1 Hz at open-circuit potential. The distribution of relaxation times (DRT) analysis was computed from EIS data using the open-source MATLAB toolbox “DRTtools,” developed by Prof. Francesco Ciucci’s research group [43].

### Material Characterizations

Nuclear magnetic resonance (NMR) measurements were performed on Avance NEO 400 MHz (Bruker, Germany). Raman spectra (Horiba LabRAM ARAMIS) were used to detect the solvent structures at the electrolyte/Zn interface. The morphology was examined using a field-emission scanning electron microscope (SEM, Hitachi Regulus 8230). X-ray photoelectron spectroscopy (XPS) analysis were carried out ESCALAB 250XI (Thermo Scientific) spectrometer to investigate the chemical states of the elements. Time-of-flight secondary-ion mass spectrometry (TOF-SIMS) was utilized to measure the three-dimensional morphology and two-dimensional distribution of cycled Zn in different electrolytes. In situ optical tests were performed using an optical microscope.

### Simulation Methods

The first-principles density functional theory calculations for adsorption energies and permanent dipole moments were performed using the Vienna Ab initio Simulation Package (VASP) code. The electrostatic surface potential (ESP) maps, along with the highest occupied molecular orbital (HOMO) and lowest unoccupied molecular orbital (LUMO), were generated using GaussView 6.0 software based on the converged self-consistent field (SCF) electron density. All calculations were performed using the Perdew–Burke–Ernzerhof (PBE) exchange–correlation functional.

In molecular dynamics (MD) simulations, two simulation models were developed to compare the ability of *L*-CN to participate in solvation shell formation. In the first model, 1100 water molecules and 40 ZnSO_4_ molecules were randomly placed inside a cubic box of 33.1 Å × 33.1 Å × 33.1 Å. _4_. In the second model, the electrolyte with the additive included the standard electrolyte along with 5 *L*-CN molecules. Initially, the energy of each system was minimized using the steepest descent algorithm. Following the equilibration phase, a 10 ns production run was conducted under the Constant Number, Volume, Temperature (NVT) ensemble at 298.15 K and 1 atm. Then, the system was equilibrated under the Constant Number, Pressure, Temperature (NPT) ensemble for 10 ns. The simulations were carried out with a time step of 1 fs.

The Zn(002) surface was modeled using a slab model with the (4 × 4) surface unit cell. During the geometry optimization, the bottom two tri-layers were fixed, while the top two tri-layers were fully relaxed. To prevent the artificial interaction between the slab and its neighboring images, a vacuum layer of more than 15 Å was added along the *z*-axis. The 3 × 3 × 1 Monkhorst–Pack k-point grid was used for sampling the Brillouin zone. The convergence criteria for the energy in the self-consistent field calculation and the residual force in the geometry optimization were set at 1 × 10^−6^ eV and 0.03 eV Å^−1^, respectively. The adsorption energy was defined via Eq. 1:

1 $$E_{{{\mathrm{ads}}}} = E_{{{\mathrm{system}}}} - E_{{{\mathrm{surface}}}} - E_{{{\mathrm{molecule}}}}$$

where *E*_ads_, *E*_system_, *E*_surface_, and *E*_molecule_ represent the adsorption energy between molecule and Zn surface, the total energy of the system with an adsorbed molecule, the total energy of the Zn surface slab model, and the total energy of an isolated molecule, respectively.

## Results and Discussion

### Simulations on Ion–Dipole Interactions and Solvation Structures

**Fig. 2:**
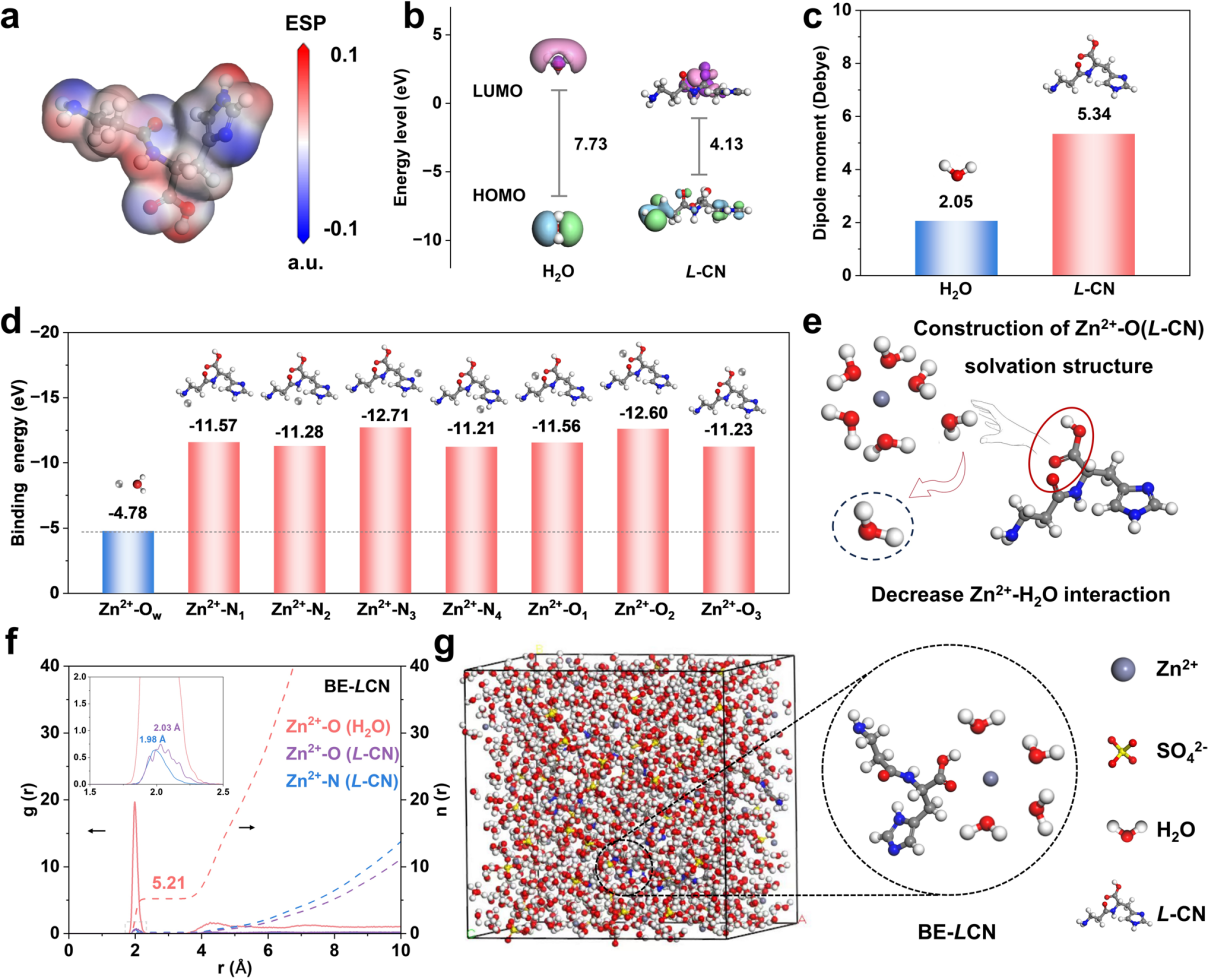
Coordination chemistry and solvation structures in various electrolytes. **a** ESP mapping of the *L*-CN additive. **b** HOMO and LUMO energy of H_2_O and *L*-CN. **c** Dipole moments of H_2_O and *L*-CN. **d** Binding energy between Zn^2+^ and *L*-CN in different sites. **e** Schematic of *L*-CN accelerating the Zn(H_2_O)_6_^2+^ dissociation via ion–dipole interactions. **f** Radial distribution functions of different sites in BE-*L*CN. **g** 3D snapshot of the *L*-CN obtained from simulations

To break down the ion–dipole interaction for achieving accelerated desolvation kinetics, it is ideal to introduce the electrolyte additive with numerous hydrophilic groups to disturb the local structure of Zn^2+^–H_2_O [44, 45]. As a natural dipeptide, *L*-CN is formed by *β*-alanine and *L*-histidine through a peptide bond, featuring multiple electron-polar groups such as carboxyl (–COOH), amino (–NH_2_), carbonyl (–CO), and imidazole groups (Fig. S1). Density functional theory (DFT) calculations combined with electrostatic potential (ESP) mappings clearly reveal the charge distribution of *L*-CN in Fig. 2a. The regionalized distribution of strongly electronegative atoms (O/N) and weakly electronegative atoms (C/H) leads to a pronounced separation of the charge centers. Moreover, the *π*-electron around the imidazole group possesses electron delocalization [46], exhibiting permanent dipole moment [47]. Frontier molecular orbital analysis reveals that *L*-CN exhibits a reduced highest occupied molecular orbital (HOMO) and the lowest unoccupied molecular orbital (LUMO) gap (4.13 eV) compared to H_2_O (7.73 eV) (Fig. 2b), indicating a more favorable electronic structure for further reaction [48]. As a result, the dipole moment of *L*-CN (5.34 D) surpasses that of H_2_O (2.05 D) (Fig. 2c), about 2.5 times higher than the pure solvent. The classical equation of ion–dipole interactions is according to Eq. 2:

2 $$E_{{{\mathrm{ion}} - {\mathrm{dipole}}}} = - \frac{{Z\mu {\mathrm{cos}}\alpha }}{{r^{{2}} }}$$

The higher dipole moment of *L*-CN is expected to interact with Zn(H_2_O)_*x*_^2+^ groups to accelerate the initial ion–dipole dissociation of Zn(H_2_O)_*x*_^2+^ to release plentiful free Zn^2+^ [49], where *Z*, *µ*, *r*, and *α* represent the ionic charge, permanent dipole moment of the molecule, the distance from the ion to the center of the dipole, and the dipole angle, respectively [50].

Further, the binding energies of Zn^2+^ with the N_1_, N_2_, N_3_, N_4_, and O_1_, O_2,_ O_3_ sites in *L*-CN were specified, which are higher than that of O_*w*_ in H_2_O (Fig. 2d). Moreover, the N_1_, N_3_, O_1_, and O_2_ sites exhibit higher binding energies, which help to remodel the Helmholtz layer, enabling *L*-CN to competitively displace H_2_O molecules from [Zn(H_2_O)_6_]^2+^ and weaken the Zn^2+^–H_2_O coordination. As long as the Zn^2+^–H_2_O bond is cleaved, high-energy Zn^2+^ and solvated H_2_O can independently escape with the replacement of *L*-CN (Fig. 2e). To further investigate the solvation shell information, the molecular dynamics (MD) simulations were performed, as shown in Figs. 2f, g and S2. In pure ZnSO_4_-based aqueous electrolyte, the peak at 1.97 Å with a coordination number of 5.44 corresponds to the Zn^2+^–O(H_2_O). The peak intensity of the Zn^2+^–O(H_2_O) was reduced with the addition of *L*-CN, and the coordination number decreases to 5.21. Moreover, two kinds of Zn^2+^–N(*L*-CN) and Zn^2+^–O(*L*-CN) peaks were observed at 1.98 and 2.03 Å, demonstrating the reconstruction of the Zn^2+^(H_2_O)_*x*_(*L*-CN)_*y*_. Calculations based on mean square displacement (MSD) reveal that the self-diffusion coefficient of Zn^2+^ in BE-*L*CN rises to 2.70 × 10^−6^ cm^2^ s^−1^ compared with BE (2.11 × 10^−6^ cm^2^ s^−1^) (Fig. S3), indicating that the reconstructed Zn^2+^(H_2_O)_*x*_(*L*-CN)_*y*_ structure facilitates rapid ion transport [51, 52].

### Remodeling of Hydrogen Bonding and Helmholtz Plane Structure

**Fig. 3:**
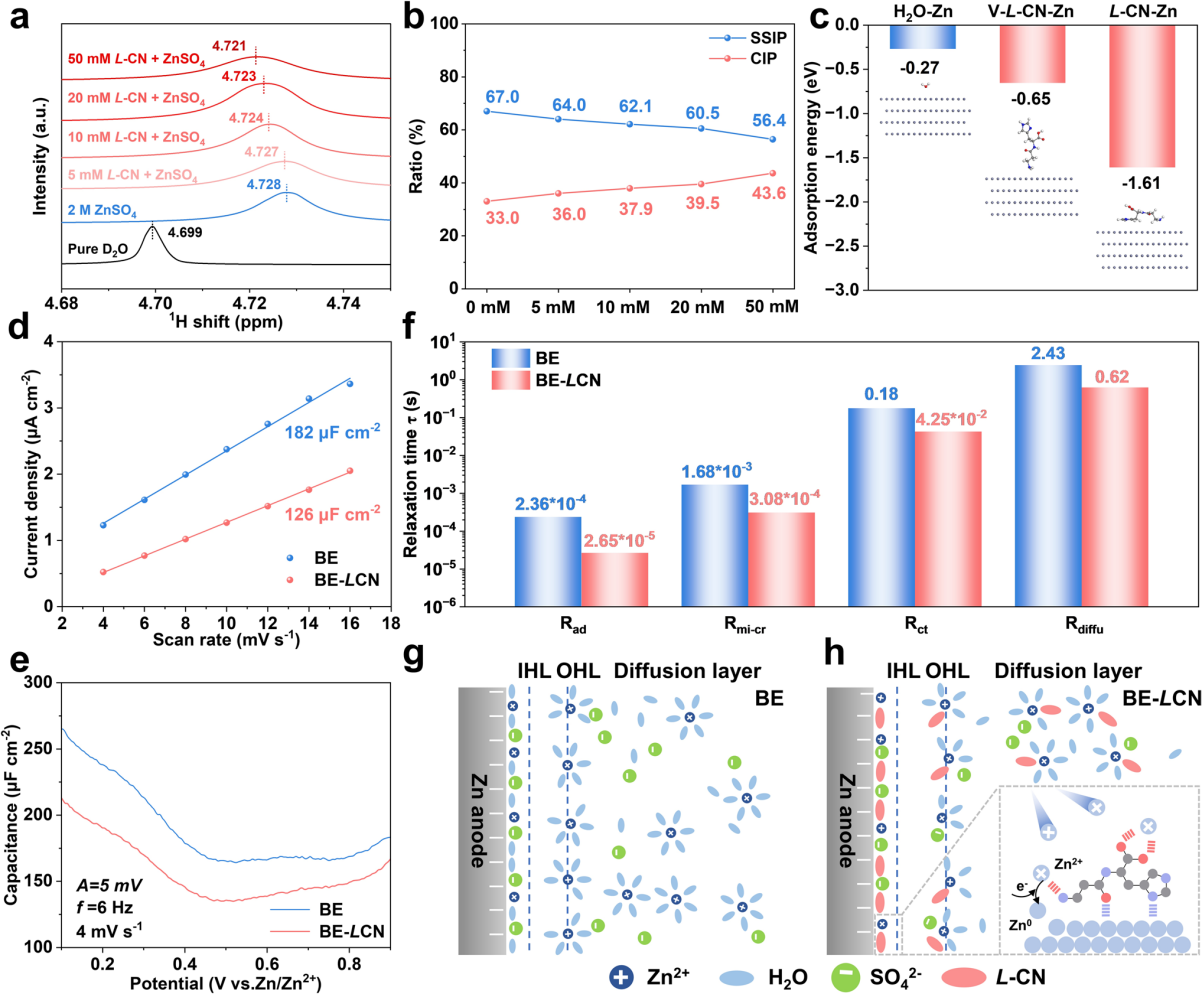
Remodeling of hydrogen bonding and the Helmholtz plane structure in various electrolytes. **a** NMR of different electrolytes. **b** Raman shift of the electrolytes containing different concentrations of *L*-CN. **c** The DFT calculation of the adsorption energy of H_2_O, vertical-*L*-CN, and *L*-CN on Zn (002) plane. **d** Calculated EDLC and **e** ACV measurement for cells with BE and BE-*L*CN. **f** Summary of relaxation times of *R*_ad_, *R*_mi-cr_, *R*_ct_, and *R*_diffu_ after 60 min plating. Schematic illustrations of the Helmholtz plane layer for Zn electrode in **g** BE and **h** BE-*L*CN

To further explore the functions of *L*-CN in regulating Zn^2+^–H_2_O behaviors, the ^1^H nuclear magnetic resonance (NMR) was used to elucidate the different hydrogen-bond (HB) networks. As illustrated in Fig. 3a, upon the addition of 2 M ZnSO_4_ to the pure D_2_O, a significant downfield shift was observed. Conversely, the downshift and wide peak of the ^1^H chemical shifts with the gradually increasing content of *L*-CN indicate increased electron delocalization, thereby reconstructing the solvation shell and altering the HB network [53]. In detail, the O–H stretching vibration peak in Raman spectra was deconvoluted into strong, medium, and weak O–H bonds (Fig. S4), respectively [54]. With increasing concentration of *L*-CN, the proportion of strong H-bonds gradually decreased from 50.7% to 40.3%, indicating a significant disruption of the strong HB network in the solvation shell. Further analysis of the *ν*-SO_4_^2−^ vibration was carried out, and two ion-pair species were deconvoluted as the solvent-separated ion pair (SSIP, [Zn^2+^(H_2_O)_6_·SO_4_^2−^]) and the contact ion pair (CIP, [Zn^2+^(H_2_O)_5_·OSO_4_^2−^]) [55]. Notably, with the increasing concentration of *L*-CN, the ratio of CIP progressively increases (from 33.3% to 43.3%) (Figs. 3b and S5), confirming the anion chemistry and behaviors were changed [56].

The concentration effect of *L*-CN in the basic 2M ZnSO_4_ electrolyte (BE) was evaluated. At the concentration of 10 mmol L^−1^, the lifespan is extended with the lowest overpotential (Fig. S6), which is also verified by ionic conductivity (Fig. S7). Thus, the concentration of *L*-CN in the BE is set as 10 mmol L^−1^ (noted as BE-*L*CN). With the addition of *L*-CN, the optimal solvation structure delivered a smaller activation energy than that of BE (27.93 vs. 46.14 kJ mol^−1^) (Fig. S8) [57], as well as the increased Zn^2+^ transference number from 0.283 in BE to 0.574 in BE-*L*CN (Fig. S9), confirming rapidly dissociating the ion–dipole interactions for fast Zn^2+^ transport through its numerous electron–polar groups. As shown in Fig. 3c, the lowest adsorption energy of *L*-CN on the Zn surface (− 1.61 eV) demonstrates the preference on Zn surface via the π-electron delocalization of the imidazole group [58, 59]. Contact angle measurements were further confirmed the adsorption of electrolytes on the Zn surface (Fig. S10). The BE-*L*CN electrolyte exhibits a lower contact angle (75.14°) than BE (89.13°), attributed to the zincophilic organic functional groups in *L*-CN that improve electrolyte wettability. In order to reveal solvation shell changes, cyclic voltammetry (CV) tests were adopted to measure the electric double layer capacitance (EDLC) of Zn//Zn symmetric cells with different electrolytes (Figs. 3d and S11). Attributed to the reconstruction with the *L*-CN molecules participating in the IHP, the EDLC decreased from 182 to 126 μF cm^−2^. Similarly, alternating current voltammetry (ACV) tests confirmed that the *L*-CN can reconfigure the EDL over a range of applied potentials (Fig. 3e) [60]. The distribution of relaxation times (DRT) method was applied to accurately characterize the electrochemical processes involved in Zn^2+^ deposition. Four distinct peaks were identified, assigned to the adsorption of Zn^2+^ (*R*_ad_), migration and crystallization of Zn^2+^ across interface (*R*_mi-cr_), the charge transfer process of Zn^2+^ (*R*_ct_), and the diffusion of Zn^2+^ (*R*_diffu_) [61], respectively. As exhibited in Fig. S12, the BE-*L*CN exhibits a significantly reduced *R*_ad_ compared to BE. With the prolonged plating time, the BE exhibits higher impedance magnitudes and greater variations, indicating interfacial instability [62]. In contrast, the BE-LCN exhibits a significantly reduced *R*_ad_, indicating that *L*-CN effectively facilitates the desolvation of hydrated Zn^2+^ as it approaches the electrode interface, thereby promoting subsequent charge transfer. The *R*_mi-cr_ and *R*_ct_ of the BE-LCN system remain low and stable. Notably, the suppression of *R*_diffu_ in BE-LCN indicates mitigated concentration polarization and improved Zn^2+^ transport toward the electrode interface. Meanwhile, the cell with BE-*L*CN shows significantly lower relaxation times (*τ*), reflecting an enhanced kinetics (Fig. 3f). As summarized in Fig. 3g, h, the ion–dipole interactions in the Helmholtz layer were reconstructed due to the employment of a high permanent dipole moment of *L*-CN proposed from the electrolyte to the interface. In the diffusion layer, *L*-CN enters the primary solvation shell and displaces water molecules to form a crowded Zn^2+^-conductive structure, facilitating rapid Zn^2+^ desolvation for subsequent deposition. Meanwhile, the preferential adsorption on the Zn surface via the π-electron system and the local deposition electric field have been altered for further smooth plating to inhibit dendrite growth [63].

### Monitoring Zn Deposition Behavior

**Fig. 4:**
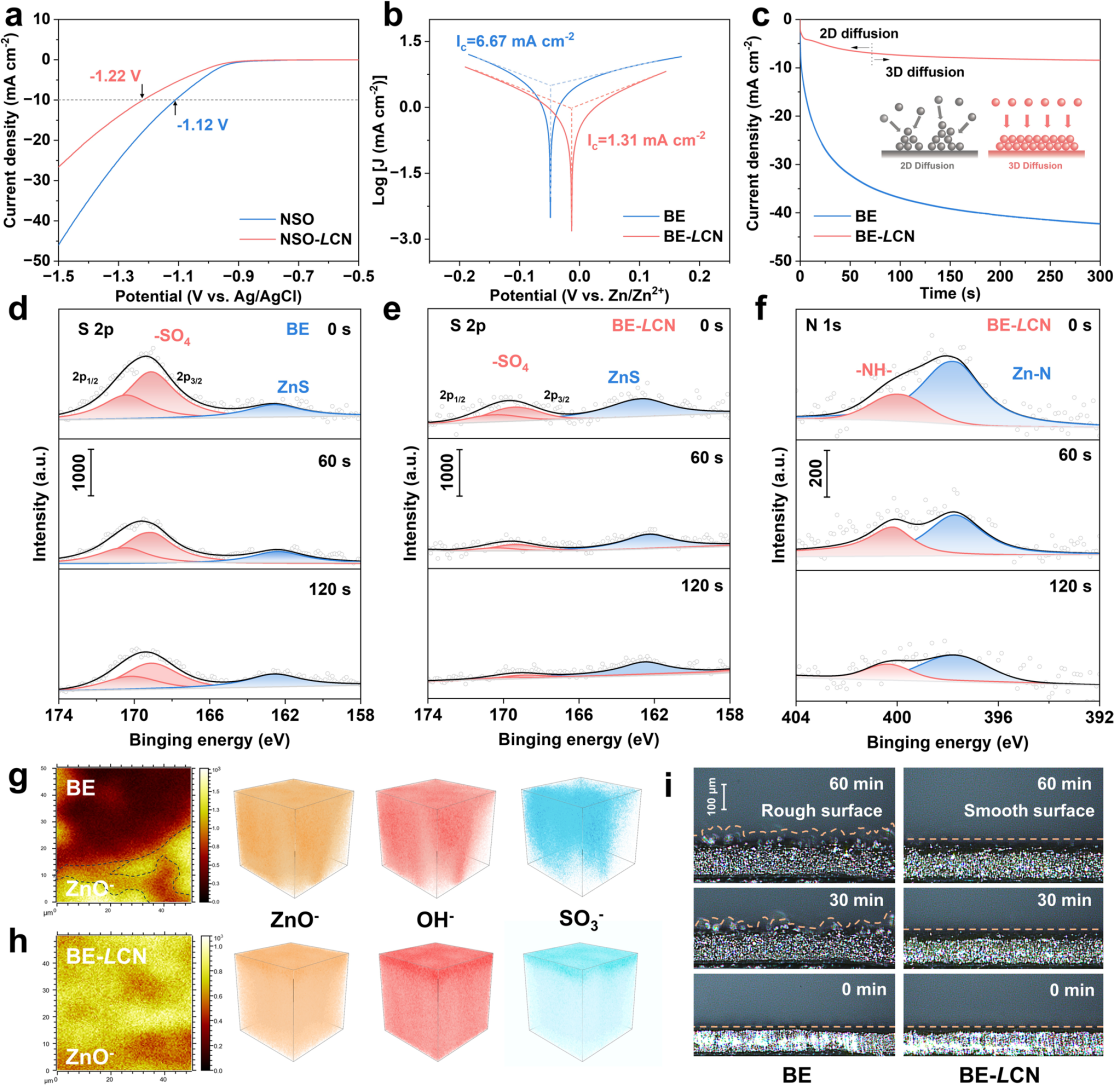
Interfacial dynamics and Zn deposition behavior. **a** LSV curves of Zn electrodes in NSO and NSO-*L*CN. **b** Tafel test and **c** CA test of Zn//Zn cells in BE and BE-*L*CN. In-depth XPS spectra of Zn anode after 20 cycles in different electrolytes with sputtering times of 0, 60, and 120 s: **d** S 2*p* in BE; **e** S 2*p* in BE-*L*CN; **f** N 1*s* in BE-*L*CN. 2D variation of ZnO^−^ and spatial distribution of ZnO^−^, OH^−^, and SO_3_^−^ in **g** BE and **h** BE-*L*CN after 20 cycles. **i** In situ optical microscopy observations of Zn^2+^ deposition in BE and BE-*L*CN

Successively, the effect on inhibiting the formation of active water molecules in the solvation shell was investigated. By lowering the cathodic current density and shifting the HER potential from − 1.12 to − 1.22 V (Fig. 4a), HER suppression is achieved by the remodeling of solvation shell. Meanwhile, the Tafel corrosion curves reveal that the corrosion current density of the BE-*L*CN (1.31 mA cm^−2^) is significantly lower than that of the BE (6.67 mA cm^−2^) (Fig. 4b). To further validate the effectiveness of BE-*L*CN, an overpotential of − 150 mV (Fig. 4c) was applied to the Zn anode, tracing the plating evolution. In the BE, the Zn plating behavior tends to be an uncontrolled Zn^2+^ two-dimensional (2D) diffusion process. While with the reconstruction of solvation shell, a stable current density is observed at the Zn electrode, indicating a transition to three-dimensional (3D) diffusion with more uniform deposition [64]. The investigation on Zn nucleation under different current densities provides in-depth insights (Fig. S13). In BE system, with the increased current density, randomly oriented Zn deposition and larger hexagonal crystalline grains are observed, which are consistent with classical electrocrystallization theory. On the contrary, the more favored (002) texture initiates since the nucleation stage with the help of *L*-CN coating at 1 mA cm^−2^. Moreover, the sizes of hexagonal crystalline platelets decrease dramatically as current density increases. The results show that *L*-CN enables delocalization and lateral growth of Zn atoms.

The plating morphologies of Zn metal at 1 mA cm^−2^ were characterized by scanning electron microscope (SEM) (Fig. S14). The Zn surface under BE displays extensive dendrites and significant petal-like particles along with serious aggregation. In contrast, the uniform and dense Zn surface morphology was achieved under the *L*-CN assisted solvation shell. As shown in Fig. 4d, two prominent SO_4_^2−^ peaks centered around 169.1 and 170.5 eV, and a strong ZnS peak close to 162.6 eV in the high-resolution S 2*p* spectra are observed [65]. In detail, the content of the sulfur-containing species persists continuously throughout the etching from 0 to 120 s. In contrast, the cycled Zn anode in BE-*L*CN exhibited substantially attenuated signals of sulfur-containing species with increasing sputtering depth, confirming effective suppression of HER and highlighting the role of the *L*-CN in preventing the interface side reactions (Fig. 4e). Furthermore, X-ray diffraction (XRD) patterns of Zn foils after immersion confirm that there is no by-product (ZnSO_4_(OH)_6_·4H_2_O) occurring using BE-*L*CN compared to BE (Fig. S15). Notably, BE-*L*CN induces fewer by-products than BE due to its near-neutral pH (Fig. S16). Furthermore, the N 1*s* spectra characteristic peaks at 400 and 398.8 eV are assigned to –NH– and Zn–N bonds [66, 67], further confirm the reaction between *L*-CN and the Zn surface (Fig. 4f). TOF–SIMS analysis provides further insights into the 3D morphological reconstruction and species distribution. Under the BE, the Zn anode exhibited pronounced elemental gradients and structural defects, characterized by highly uneven distributions of ZnO^−^, OH^−^, and SO_3_^−^ (Figs. 4g and S17). A thin layer is observed after the addition of *L*-CN (Fig. 4h), indicating that the introduction of *L*-CN is beneficial to reconstruct the IHP layer and repulse H_2_O. The morphological plating evolution of the Zn was monitored by in situ optical microscopy in these two electrolytes (Fig. 4i). In BE, irregular protrusions appeared on the Zn surface after 30 min of deposition and rapidly developed into sharp dendrites within 60 min. With the solvation regulation by *L*-CN, the thickness of the deposited Zn layer increased uniformly, and no obvious dendrites and HER bubbles were observed, demonstrating the excellent dendrite-suppressing function and Zn^2+^ deposition-regulating capability of *L*-CN.

### Study of Electrochemical Behavior

**Fig. 5:**
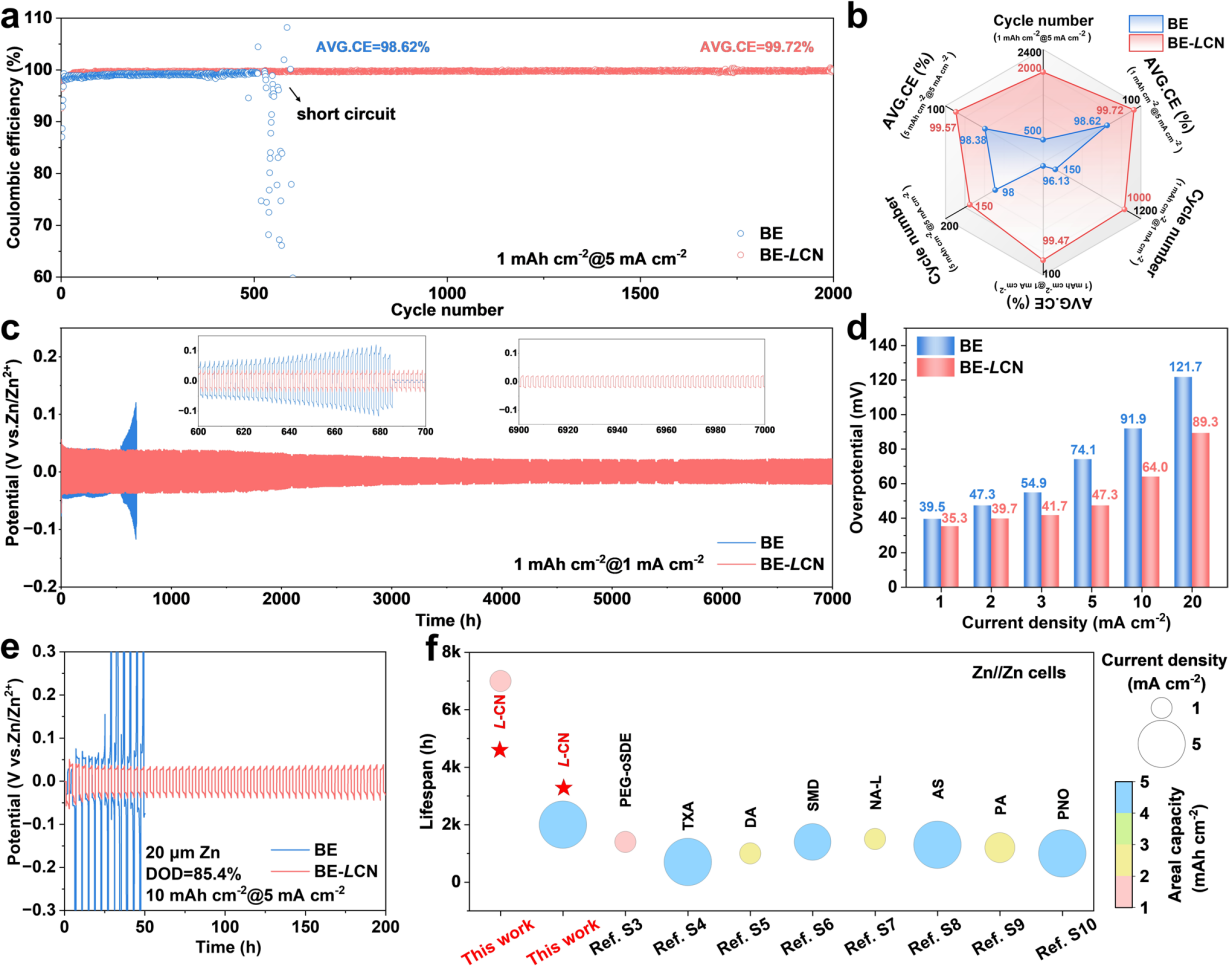
Electrochemical performance of symmetric and asymmetric cells. **a** CE of Zn//Cu cells in BE and BE-*L*CN at 5 mA cm^−2^. **b** Summary of average CE and cycle performance for Zn//Cu cells at various test conditions. **c** Long-term cycling stability of symmetric cells in BE and BE-*L*CN at 1 mA cm^−2^. **d** Overpotentials of cells with/without *L*CN at different current densities. **e** Long-term performance of Zn//Zn cells in BE and BE-*L*CN with high DOD of 85.4%. **f** Performance comparison of symmetrical cells with different electrolyte additives between previous studies and this work

The Zn//Cu asymmetric cells were assembled based on two electrolytes to evaluate the reversibility of Zn plating/stripping process. The Zn//Cu cell with BE exhibited a short cycling lifespan with a lower average CE (98.62%) within 500 cycles (Figs. 5a and S18). In contrast, benefiting from reconstructed ion–dipole behaviors in the Helmholtz layer, the cells with BE-*L*CN exhibited a stable voltage profile and a high average CE of 99.72% over 2000 cycles. Even the current was decreased to 1 mA cm^−2^ or the plating capacity increased to 5 mAh cm^−2^, similar comparative trends were observed (Figs. 5b, S19 and S20). Galvanostatic intermittent titration technique (GITT) measurements further confirm that the fast Zn^2+^ desolvation and diffusion capabilities of BE-*L*CN reduce concentration polarization from 5.92 to 4.41 mV, thereby promoting uniform Zn^2+^ deposition (Fig. S21) [68].

In the long-term cycling test, the symmetric cells in BE failed after 680 h, exhibiting a sharp overpotential rise and decrease. While the *L*-CN-modified symmetric cells demonstrated outstanding stability for over 7000 h with stable overpotential, almost 10 times longer than that in the BE (Fig. 5c). The degrees of calendar aging were also assessed. Obviously, the Zn anode underwent severe passivation due to the accumulation of by-products in the BE, and the cell failed after 100 cycles (Fig. S22). In contrast, the reconfigured IHP layer inhibited the active water molecules form triggering the HER, maintaining interfacial stability throughout multiple rest periods. EIS measurements further verify the cycling stability of BE-*L*CN (Fig. S23). During 50 cycles, the BE-*L*CN system showed no significant increase in interfacial contact resistance. In contrast, the impedance in BE system rises continuously with cycling, indicating progressive deterioration of the electrode/electrolyte interface. Furthermore, the rate performance of the Zn//Zn cells with current densities increasing from 1 to 20 mA cm^−2^ was investigated (Figs. 5d and S24). The BE-*L*CN demonstrates significantly reduced voltage hysteresis across a range of current densities, and it is only 90 mV under 20 mA cm^−2^. To evaluate the deep cycling capability, Zn//Zn symmetric cells were tested under different DODs of 40% and 85.4% (Figs. 5e and S25), respectively. Notably, the symmetric cells within BE failed within 10 cycles, accompanied by a significantly up-and-down polarization voltage. In contrast, the *L*-CN-modified cell maintained stable cycling for 800 h at 40% DOD and 200 h at 85.4% DOD. As summarized in Fig. 5f and Tables S2–S3, the long-term cycling performance is superior to most of the modified electrolytes reported, exhibiting *L*-CN with a remarkable ability to break down the ion–dipole interactions and change interfacial desolvation–diffusion behavior near the IHP layer of the EDL, improving the stability of Zn anodes.

### Electrochemical Performance of Full Cells

**Fig. 6:**
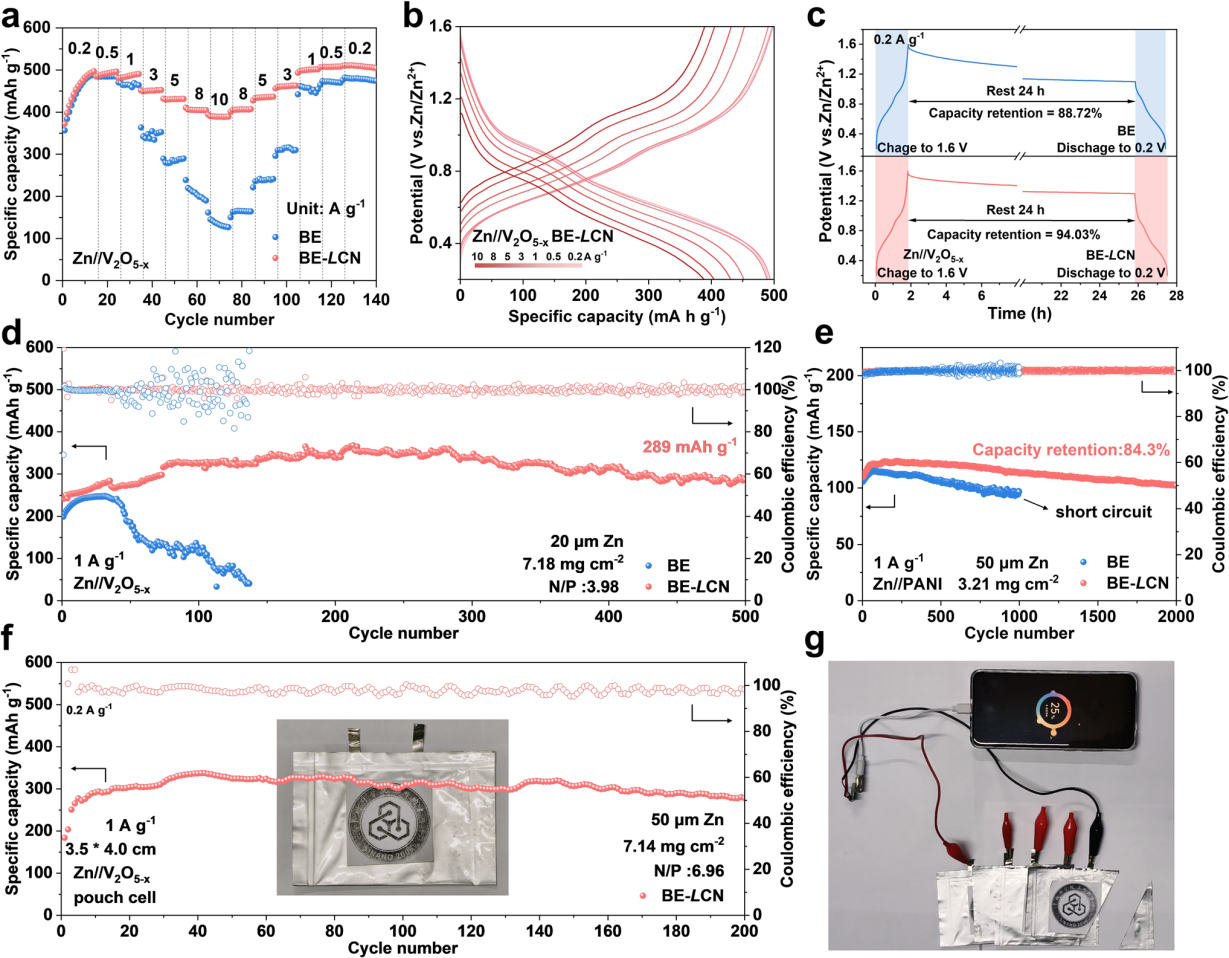
Full-cell performance of Zn//V_2_O_5−*x*_ and Zn//PANI cells. **a** Rate performance of Zn//V_2_O_5−*x*_ full cells in BE and BE-*L*CN. **b** Capacity-voltage curves of Zn//V_2_O_5−*x*_ full cell in BE-*L*CN at different current densities. **c** Self-discharge profiles of Zn//V_2_O_5−*x*_ full cells in BE and BE-*L*CN. **d** Long-term cycling performance of Zn//V_2_O_5−*x*_ full cells in BE and BE-*L*CN at 1 A g^−1^ (20 µm Zn, N/P: 3.98). **e** Long-term cycling performance of Zn//PANI full cells in BE and BE-*L*CN at 1 A g^−1^. **f** Cycling performance of pouch cell at 1 A g^−1^ and **g** photograph of pouch cells for empowering a phone

To validate the practical application, the electrochemical performance of full cells assembled with V_2_O_5−*x*_ and polyaniline (PANI) cathodes was systematically evaluated. The CV curves of Zn//V_2_O_5−*x*_ cell with BE and BE-*L*CN exhibited similar redox peaks, but the incorporation of *L*-CN led to narrowed voltage gaps (Fig. S26), indicating fast desolvation between Zn^2+^ and H_2_O in the BE-*L*CN. Moreover, the *L*-CN-modified Zn//V_2_O_5−*x*_ full cell delivers high reversible rate capacities from 499 to 388 mAh g^−1^ when shifting current rate from 0.5 to 10 A g^−1^, respectively (Figs. 6a, b and S27). Under 10 A g^−1^, the cell with BE-*L*CN operates much better than that in BE (388 vs. 125 mAh g^−1^). The *L*-CN-modified Zn//V_2_O_5−*x*_ cells retains 94.03% of its initial capacity after 24 h, outperforming the BE (88.72%) (Fig. 6c). In the Zn//PANI full cell, a similar comparative trend was detected. The CV curves, rate performance, and self-discharge suppression of the *L*-CN modified Zn//PANI cell are effectively enhanced (Figs. S28–S30), demonstrating the universal applicability of *L*-CN additives in improving capacity and stability.

The long-term cycling performance of the two cathode systems is presented in Figs. S31–S38. As expected, both the *L*CN-modified Zn//PANI and Zn//V_2_O_5−*x*_ full cells exhibited significantly lower electrochemical impedance before and after cycling, and the cycled cathode surface remained smooth and flat. The *L*-CN-modified Zn//V_2_O_5−*x*_ full cell affords a specific capacity of 444 mAh g^−1^ over 500 cycles at 1 A g^−1^, whereas the cells in pristine electrolyte only achieved a specific capacity of 296 mAh g^−1^. At 10 A g^−1^, the cell delivered stable cycling performance with a high reversible capacity of 400 mA h g^−1^ after 4000 cycles. Even at 0.5 A g^−1^, the cell maintained stable cycling, with a high capacity retention of 95.2% after 300 cycles. Similarly, the *L*-CN-modified Zn//PANI full cells demonstrate outstanding long-term performance, achieving the capacity retention of 86.3% at 3 A g^−1^ after 4000 cycles, much better than previously reported systems (Table S4). For practical applications, the limited Zn source with high mass loading of cathode is to achieve a lower negative/positive ratio (N/P ratio) [69]. The specific capacity of the Zn//V_2_O_5−*x*_ cell in BE-*L*CN operated stably for 500 cycles (Fig. 6d), much longer than that with BE (50 cycles). Similarly, the Zn//PANI full cells (Fig. 6e) also achieved a high capacity retention of 84.3% after 2000 cycles. In a large-sized pouch cell with 7.14 mg cm^−2^ and a low N/P ratio of 6.96 (Fig. 6f), the cell exhibited exceptional cycling stability over 200 cycles at 1 A g^−1^ with the capacity retention of ~ 100%, successfully empowering a smart electronic device (Fig. 6g) [70]. This performance is much better than previously reported works (Table S5).

## Conclusions

In summary, a novel concept of remodeling Helmholtz plane by significantly regulating ion–dipole interactions has been proposed to enhance the Zn(H_2_O)_6_^2+^ desolvation kinetics and Zn ion/atom diffusion kinetics by introducing multi-functional molecular additive, thereby achieving durable Zn deposition without dendritic growth. Both theoretical simulations and spectroscopic analysis reveal that the higher dipole moment of representative *L*-CN preferentially interacts with Zn^2+^, weakening the coordination between Zn^2+^ and H_2_O and facilitating the Zn^2+^ desolvation process. Besides, the preferential adsorption of *L*-CN on the Zn surface further remodels the IHP layer with abundant Zn^2+^ flux. Consequently, the *L*-CN-modified cells enable a long lifespan up to 7000 h, a high CE of 99.72%, and even remain stable for over 200 h under a high DOD of 85.4%. The excellent stability and rate capability are also demonstrated by Zn//V_2_O_5−*x*_ full cells with high capacity of 289 mAh g^−1^ after 500 cycles at 1 A g^−1^ under a low N/P ratio of 3.98. Impressively, the large-scale pouch cell employed with *L*-CN operates stably for hundreds of cycles and powers the smart devices, highlighting the benefit of remodeling Helmholtz plane for practical applications.

## Supplementary Information

Below is the link to the electronic supplementary material.

Supplementary file1 (DOCX 7192 KB)
