# Supplementary material for: Decoupling Ion–Dipole Interactions via Competitive Coordination for Durable Dendrite-Free Zinc Metal Batteries
Source: Nanomicro Lett. 2026 Aug 10;19:24. doi: 10.1007/s40820-026-02306-5 (PMC13454087; doi:10.1007/s40820-026-02306-5)
Supplement: Supplementary file 1 [file 40820_2026_2306_MOESM1_ESM.docx]

Supporting Information for

**Decoupling Ion-Dipole Interactions via Competitive Coordination for Durable Dendrite-free Zinc Metal Batteries**

Bixian Chen^1, 4#^, Xiaomin Cheng^1, 8#^, Xiang Li^5^, Qinghua Guan^1^, Hao Li^2, 3^, Yongzheng Zhang^6^, Shuang Cheng^1^, Huihua Li^2, 3^, Yunjian Liu^4,^ *, Jing Zhang^7^, Hongzhen Lin^1,^ *, Jian Wang^2, 3,^ *

^1^ *i*-Lab & CAS Key Laboratory of Nanophotonic Materials and Devices, Suzhou Institute of Nano-Tech and Nano-Bionics, Chinese Academy of Sciences, Suzhou 215123, P. R. China

^2^ Helmholtz Institute Ulm (HIU), Ulm D89081, Germany

^3^ Karlsruhe Institute of Technology (KIT), Karlsruhe D76021, Germany

^4^ School of Materials Science and Engineering, Jiangsu University, Zhenjiang 212013, P. R. China

^5^ Anhui Provincial Key Laboratory of Green Carbon Chemistry, Fuyang Normal University, Fuyang 236037, P. R. China

^6^ School of Textile & Clothing, Nantong University, Nantong 226019, P. R. China

^7^ School of Materials Science and Engineering, Xi’an University of Technology, Xi’an 710048, P. R. China

^8^ Guangdong Institute of Semiconductor Micro-Nano Manufacturing Technology, Guangdong 518103, P. R. China

^#^ Bixian Chen and Xiaomin Cheng contributed equally to this work.

*Corresponding authors. E-mail: lyjian122331@ujs.edu.cn (Yunjian Liu); hzlin2010@sinano.ac.cn (Hongzhen Lin); wangjian2014@sinano.ac.cn or jian.wang@kit.edu (Jian Wang)

**S1 Supplementary Figures**

**Fig. S1** Schematic diagram of the *L*-CN molecular structure.

**Fig. S2** **a** Radial distribution functions of Zn^2+^-O (H_2_O) in BE. **b** The 3D snapshot of the BE obtained from MD simulations.

**Fig. S3** MSD of Zn^2+^ in different electrolytes.

**Fig. S4** Fitted Raman spectra of *ν*-OH in **a** 0 mmol L^-1^ *L*-CN, **b** 5 mmol L^-1^ *L*-CN, **c** 10 mmol L^-1^ *L*-CN, **d** 20 mmol L^-1^ *L*-CN, and **e** 50 mmol L^-1^ *L*-CN. **f** The summary proportions of strong, medium, and weak H-bonds in the systems.

**Fig. S5** Fitted Raman spectra of *ν*-OSO_3_ in **a** 0 mmol L^-1^ *L*-CN, **b** 5 mmol L^-1^ *L*-CN, **c** 10 mmol L^-1^ *L*-CN, **d** 20 mmol L^-1^ *L*-CN, and **e** 50 mmol L^-1^ *L*-CN.

**Fig. S6** The long-term cycling performance with *L*-CN under various concentrations (0-50 mmol L^-1^).

**Fig. S7** Ionic conductivity test with *L*-CN under different concentrations (0-50 mmol L^-1^).

The ionic conductivities (*σ*) were tested by two blocking electrodes (SS) and calculated according to equation S1:

$\sigma=\frac{L}{RS}$	(S1)

where *L* represents the diffusion thickness (1.624 mm), and *S* is the contact area (0.503 cm^-2^) between SS and electrolyte, R_0_=5.709 Ω, R_5_=5.857 Ω, R_10_=5.901 Ω, R_20_=6.175 Ω, R_50_=6.437 Ω.

**Fig. S8** Nyquist plots of symmetric cells employed with **a** BE and **b** BE-*L*CN in at different temperatures. **c** Charge transfer activation energies based on EIS impedance. **d** Equivalent circuit diagram used for EIS fitting.

The charge transfer activation energies (*E_a_*) were calculated by fitting to the Arrhenius equation:

$\frac{1}{R_{ct}}=A\exp\left( - \right.\frac{E_{a}}{RT})$	(S2)

Where *R_ct_* represents the charge-transfer resistance, *E_a_* denotes the activation energy, *R* is the ideal gas constant, and T represents the temperature in Kelvin.

**Fig. S9** The Zn^2+^ transference number (*t_Zn_^2+^*) of **a** BE and **b** BE-*L*CN. **c** The *t_Zn_^2+^* of various electrolytes.

The t_Zn_^2+^ is evaluated by the equation S3:

$t_{{Zn}^{2+}}=\frac{I_{s}(\Delta V-I_{0}R_{0})}{I_{0}(\Delta V-I_{s}R_{s})}$	(S3)

Where *ΔV* is the applied voltage (20 mV), *I_0_* and *I_s_* are the initial and final current, *R_0_* and *R_s_* are the initial and final resistance.

**Fig. S10** Contact angle measurements of **a** BE and **b** BE-*L*CN electrolytes on the Zn anode.

**Fig. S11** Cyclic voltammetry (CV) tests at different scan rates in **a** BE and **b** BE-*L*CN.

The EDL capacitance was calculated through the equation S4:

$C=\frac{i_{c}}{v}$	(S4)

Where *i_c_* represents the double-layer current and *v* denotes the scan rate. The linear relationship between *i_c_* and *v* allows for the calculation of capacitance, with C derived from the slope of the *i_c_* versus *v* plot. Specifically, *i_c_* is calculated as *i_c_* =(*i_0_*^+^-*i_0_*^−^)/2, which is the average of the current difference between the forward and reverse scans at 0 V.

**Fig. S12** Operando EIS evolution and corresponding analysis of DRT during galvanostatic plating in **a** BE and **b** BE-*L*CN. EIS evolutions during plating at 1 mA cm^-2^ in **c** BE and **d** BE-*L*CN symmetric cells.

The EIS measurements were performed after Zn was deposited at 1 mA cm^-2^ for 6 min, followed by a 1 min rest period. The total Zn deposition time is 60 min.

**Fig. S13** The SEM images of Zn anodes after plating at first cycle with different current densities and 1 mAh cm^-2^: **a** 1 mA cm^-2^, **b** 2 mA cm^-2^ and **c** 5 mA cm^-2^ in BE; **d** 1 mA cm^-2^, **e** 2 mA cm^-2^ and **f** 5 mA cm^-2^ in BE-*L*CN.

**Fig. S14** The SEM images of the cycled Zn foils in **a** BE and **b** BE-*L*CN.

**Fig. S15** XRD results of Zn foil after 20 cycles at 1 mA cm^-2^, 1 mAh cm^-2^.

**Fig. S16** pH value of BE and BE-*L*CN electrolytes.

**Fig. S17** Intensity comparison of ZnO^-^, OH^-^ and SO_3_^-^ species on cycled Zn in BE and BE-*L*CN.

**Fig. S18** Capacity-voltage curves of **a** BE and **b** BE-*L*CN under different cycles.

**Fig. S19** **a** Coulombic efficiency (CE) values of Zn//Cu cells in BE and BE-*L*CN with a cut-off voltage of 0.8 V at 1 mA cm^−2^. Capacity-voltage curves of **b** BE and **c** BE-*L*CN under different cycles.

**Fig. S20** **a** CE values of Zn//Cu cells in BE and BE-*L*CN with a cut-off voltage of 0.8 V at 5 mA cm^−2^. Capacity-voltage curves of **b** BE and **c** BE-*L*CN under different cycles.

**Fig. S21** GITT profiles of symmetric cells in BE and BE-*L*CN at 1 mA cm^-2^ for 3 min and a rest of 3 min.

**Fig. S22** Resting-cycle cycling stability of symmetric cells in BE and BE-*L*CN at 1 mA cm^-2^.

**Fig. S23** EIS evolution of Zn//Zn symmetric cells during 50 cycles with **a** BE and **b** BE-*L*CN electrolytes at 1 mA cm^-2^.

**Fig. S24** Rate performances of symmetric cells in BE and BE-*L*CN.

**Fig. S25** Galvanostatic cycling performance at 11.7 mA cm^-2^, 11.7 mAh cm^-2^ under a high depth of discharge (40%) condition (Zn foil thickness: 50 μm).

The high depth of discharge (DOD) is the percentage of the capacity involved in the electrode reaction relative to the overall capacity of the Zn metal anode:

$DOD=\frac{y}{C_{Zn}*x\times{10}^{-4}}*100\%$	(S6)

where *C_Zn_* is the theoretical volume capacity of Zn (5855 mAh cm^-3^), *x* (μm) is the thickness of the Zn foil and *y* (mAh cm^−2^) represents the Zn areal capacity used in electrochemical testing.

**Fig. S26** CV curves of Zn//V_2_O_5-x_ full cells with BE and BE-*L*CN electrolytes at the second cycle.

**Fig. S27** Capacity-voltage curves of Zn//V_2_O_5-x_ full cell in BE.

**Fig. S28** CV curves of Zn//PANI full cells with BE and BE-*L*CN electrolytes at the second cycle.

**Fig. S29 a** Rate performances of Zn//PANI full cells in BE and BE-*L*CN. Capacity-voltage curves of Zn//PANI full cells in **b** BE and **c** BE-*L*CN.

**Fig. S30** Self-discharge profiles of Zn//PANI full cells in BE and BE-*L*CN.

**Fig. S31** The SEM images of the cycled V_2_O_5-x_ cathodes in **a** BE and **b** BE-*L*CN.

**Fig. S32** EIS analysis of Zn//V_2_O_5-x_ full cells in **a** BE and **b** BE-*L*CN before and after 50 cycles.

**Fig. S33** The SEM images of the cycled PANI cathodes in **a** BE and **b** BE-*L*CN.

**Fig. S34** EIS analysis of Zn//PANI full cells in **a** BE and **b** BE-*L*CN before and after 50 cycles.

**Fig. S35 a** The long-term cycling performances of Zn//V_2_O_5-x_ full cells in BE and BE-*L*CN at 1 A g^−1^. Capacity-voltage curves of Zn//V_2_O_5-x_ full cells in **b** BE and **c** BE-*L*CN.

**Fig. S36 a** The long-term cycling performances of Zn//V_2_O_5-x_ full cells in BE and BE-*L*CN at 10 A g^-1^. Capacity-voltage curves of Zn//V_2_O_5-x_ full cells in **b** BE and **c** BE-*L*CN.

**Fig. S37** The long-term cycling performances of Zn//V_2_O_5-x_ full cells in BE and BE-*L*CN at 0.5 A g^-1^

**Fig. S38** The long-term cycling performances of Zn//PANI full cells in BE and BE-*L*CN at 3 A g^-1^.

**S2 Supplementary Tables**

**Table S1** The N/P ratio of full cells

| Type of cells | Zn thickness (cm) | Zn area (cm^-2^) | Negative Capacity (mAh) | Mass Loading of Cathode (mg) | Positive Capacity (mAh) | N/P Ratio |
| --- | --- | --- | --- | --- | --- | --- |
| Coin | 0.002 | 1.13 | 13.23 | 5.64 | 3.32 | 3.98 |
| Pouch | 0.005 | 14 | 409.84 | 99.96 | 58.88 | 6.96 |

The N/P ratio is calculated by the following equation:

**Negative Capacity (mAh)** = Zn thickness (cm) * *ρ_Zn_* (g cm^-3^) * Theoretical specific capacity of Zn (mAh g^-1^) * Area of the Zn anode

**Positive Capacity (mAh)** = Mass loading of cathode (g) * Theoretical specificcapacity of V_2_O_5-x_ (mAh g^-1^)

Where ρ_Zn_, theoretical specific capacity of Zn and theoretical specific capacity of V_2_O_5_ are 7.14, 820 and 589, respectively.

**Table S2** Performance comparison of symmetrical cells with different electrolyte additives between previous studies and this work

| **Electrolyte additive** | **Current density (mA cm^-2^)** | | **Areal capacity (mAh cm^-2^)** | **Lifespan (h)** | **Reference** |
| --- | --- | --- | --- | --- | --- |
| **L-CN** | | **1** | **1** | **7000** | **This work** |
| **L-CN** | | **5** | **5** | **2000** | **This work** |
| PEG-oSDE | | 1 | 1 | 1400 | [S1] |
| TXA | | 5 | 5 | 700 | [S2] |
| DA | | 2 | 1 | 1000 | [S3] |
| SMD | | 5 | 3 | 1400 | [S4] |
| Na-L | | 2 | 1 | 1500 | [S5] |
| AS | | 5 | 5 | 1300 | [S6] |
| PA | | 2 | 2 | 1200 | [S7] |
| PNO | | 5 | 5 | 1000 | [S8] |

**Table S3** DOD and cycle lifespan comparison of symmetrical cells between previous studies and this work

| **Electrolyte additive** | **Current density (mA cm^-2^)** | | **Areal capacity (mAh cm^-2^)** | **DOD (%)** | **Lifespan (h)** | **Reference** |
| --- | --- | --- | --- | --- | --- | --- |
| **L-CN** | | **11.7** | **11.7** | **40** | **7000** | **This work** |
| **L-CN** | | **5** | **10** | **85.4** | **200** | **This work** |
| CoPcS₄ | | 10 | 3 | 51.2 | 150 | [S9] |
| NHM | | 6 | 6 | 60 | 400 | [S10] |
| CTP | | 8.8 | 8.8 | 50 | 300 | [S11] |
| PN-ps coating | | 2 | 20 | 68.3 | 400 | [S12] |
| AHRE | | - | - | 60 | 300 | [S13] |

**Table S4** Performance comparison of full cells with different electrolyte additives between previous studies and this work

| **Electrolyte additive** | **Cathode materials** | **Current**  **(A g^-1^)** | **Cycling performances** | **Reference** |
| --- | --- | --- | --- | --- |
| **L-CN** | **V_2_O_5-x_** | **1** | **500 cycles**  **95.2%**  **444 mAh g^-1^** | **This work** |
| **L-CN** | **V_2_O_5-x_** | **10** | **4000 cycles**  **93.9%** | **This work** |
| **L-CN** | **PANI** | **3** | **4000 cycles**  **86.3%** | **This work** |
| LA | NH_4_V_4_O_10_ | 5 | 2000 cycles  80.35% | [S14] |
| DP | NH_4_V_4_O_10_ | 3 | 3000 cycles  80.3% | [S15] |
| 10% Xos | ZnVO | 5 | 1000 cycles  82.9% | [S16] |
| Zn(BF_4_)_2_-AN | PANI | 1 | 2000 cycles  86.72% | [S17] |
| Glu | PANI | 1 | 1000 cycles  91% | [S18] |
| PCA-Zn | PANI | 1 | 2000 cycles  73.33% | [S19] |
| Zn(CF_3_SO_3_)_2_ | MEG/PANI | 2 | 2000 cycles  72.7% | [S20] |

**Table S5** N/P ratio and performance comparison of full cells between previous studies and this work

| **Work** | **Cathode materials** | **Type of cells** | **Cycling performances** | **N/P ratio** | **Reference** |
| --- | --- | --- | --- | --- | --- |
| **L-CN** | **V_2_O_5-x_** | **coin** | **500 cycles**  **1 A g^-1^** | **3.98** | **This work** |
| **L-CN** | **V_2_O_5-x_** | **pouch** | **200 cycles**  **1 A g^-1^** | **6.96** | **This work** |
| FCOF@Zn | MnO_2_ | coin | 200 cycles  4 mA cm^-2^ | 5 | [S21] |
| Sorbitol | CNF/MnO_2_ | coin | 500 cycles  1 A g^-1^ | 5.55 | [S22] |
| NH_4_Ac/Et_4_  NOTF | PANI | coin | 1000 cycles  1 A g^-1^ | 5~6 | [S23] |
| FPCH-ZI/Zn | MnO_2_ | coin | 600 cycles  0.5 A g^-1^ | 7.3 | [S24] |
| Zinc-plated Cu substrate | MnO_2_ | coin | 640 cycles  5 mA cm^-2^ | 5 | [S25] |
|  |  |  |  |  |  |

**Supplementary References**

[S1] Z. Shi, M. Li, X. Fu, Y. Zhang, S. Jiao et al., Bimodal Block molecule with ether-type and hydroxyl-type oxygen stabilizes Zn anode in super-dilute electrolyte. Adv. Funct. Mater. **34**(25), 2316427 (2024). <https://doi.org/10.1002/adfm.202316427>
[S2] J. Yin, H. Liu, P. Li, X. Feng, M. Wang et al., Integrated electrolyte regulation strategy: Trace trifunctional tranexamic acid additive for highly reversible Zn metal anode and stable aqueous zinc ion battery. Energy Storage Mater. **59**, 102800 (2023). <https://doi.org/10.1016/j.ensm.2023.102800>
[S3] Y. Yang, Y. Li, Q. Zhu, B. Xu, Optimal molecular configuration of electrolyte additives enabling stabilization of zinc anodes. Adv. Funct. Mater. **34**(32), 2316371 (2024). <https://doi.org/10.1002/adfm.202316371>
[S4] S. Zheng, Y. Wang, B. Luo, L. Sun, G. Duan et al., *In-situ* formation of heterogeneous interfaces inducing surface crystallographic manipulation toward highly stable Zn anode. Chem. Eng. J. **473**, 145313 (2023). <https://doi.org/10.1016/j.cej.2023.145313>
[S5] Z. Hu, F. Zhang, A. Zhou, X. Hu, Q. Yan et al., Highly reversible Zn metal anodes enabled by increased nucleation overpotential. Nanomicro Lett. **15**(1), 171 (2023). <https://doi.org/10.1007/s40820-023-01136-z>
[S6] X. Ma, Q. Wang, X. Zhang, Y. Lin, F. Zhang et al., Highly reversible zinc anode enabled by a trace-amount additive with pH buffering capability. Energy Environ. Sci. **18**(2), 982–990 (2025). <https://doi.org/10.1039/d4ee04870a>
[S7] K. Wang, H. Zhan, X.-X. Liu, X. Sun, Uncovering required molecular properties for interface regulators and modification mechanisms for Zn anode in aqueous batteries. Adv. Funct. Mater. **35**(16), 2418993 (2025). <https://doi.org/10.1002/adfm.202418993>
[S8] Q. Kang, H. Liang, Z. Zhu, F. Xing, B. Zhu et al., Non-substituted aromatic pyridine N-oxide additives with an intrinsic N→O acceptor for ultra-long-life Zn||MnO_2_ batteries. Angew. Chem. Int. Ed. **64**(10), e202420183 (2025). <https://doi.org/10.1002/anie.202420183>
[S9] Z. Fang, J. Li, L. Xiang, K. Liu, Y. Pan et al., Kinetic regulation of Zn(002) textured interfaces for highly reversible Zn–iodine batteries. Energy Storage Mater. **82**, 104592 (2025). <https://doi.org/10.1016/j.ensm.2025.104592>
[S10] L. You, S. Guo, Y. He, Y. Xie, S. Liang et al., Surface tension-derived electrical double layer modification enables practical zinc-ion pouch cells. Adv. Funct. Mater. **35**(31), 2500780 (2025). <https://doi.org/10.1002/adfm.202500780>
[S11] Z. Chen, Y. Liao, Z. Lu, G. Chen, H.M.A. Hassan et al., Amphiphilic interfacial environment reconfiguration unlocks long-life zinc-ion batteries with lean electrolytes and ultra-low N/P ratios. Angew. Chem. Int. Ed. **65**(26), e4128996 (2026). <https://doi.org/10.1002/anie.4128996>
[S12] Z. Zhang, G. Liao, Y. Lin, W. Du, Y. Zhang et al., Decoupled yet synergistic microdomains *via* *in situ* phase separation enabling ultra-stable zinc metal anodes. Adv. Energy Mater. e71136 (2026). <https://doi.org/10.1002/aenm.71136>
[S13] X. Zhang, Q. Tang, H. Luo, Y. Xu, S. Miao et al., Hydrogen bond reconstruction maneuver in eutectic electrolyte enables ultralong-lifespan zinc-ion batteries. J. Am. Chem. Soc. **147**(43), 39440–39451 (2025). <https://doi.org/10.1021/jacs.5c12021>
[S14] R. Jiang, T. Naren, Y. Chen, Z. Chen, C. Zhang et al., Enhanced hydrogen bonding through strong water-locking additives for long-term cycling of zinc ion batteries. Adv. Funct. Mater. **34**(49), 2411477 (2024). <https://doi.org/10.1002/adfm.202411477>
[S15] M. Wu, Y. Sun, Z. Yang, S. Deng, H. Tong et al., Harnessing ion-dipole interactions for water-lean solvation chemistry: achieving high-stability Zn anodes in aqueous zinc-ion batteries. Angew. Chem. Int. Ed. **63**(43), e202407439 (2024). <https://doi.org/10.1002/anie.202407439>
[S16] W. Guo, L. Xu, Y. Su, L. Zhao, Y. Ding et al., Synchronous modulation of H-bond interaction and steric hindrance *via* bio-molecular additive screening in Zn batteries. Angew. Chem. Int. Ed. **64**(5), e202417125 (2025). <https://doi.org/10.1002/anie.202417125>
[S17] Z. Zha, D. Li, T. Sun, Q. Sun, J. Hou et al., Electrolyte design *via* cation-anion association regulation for high-rate and dendrite-free zinc metal batteries at low temperature. J. Am. Chem. Soc. **146**(46), 31612–31623 (2024). <https://doi.org/10.1021/jacs.4c09524>
[S18] Y. Chen, B. Yin, Y. Zeng, H. Wang, B.-B. Xie et al., Ion-dipole-interaction-manipulated bilateral interface chemistry for deep rechargeability and high redox activity of Zn-organic batteries. Chem **11**(6), 102411 (2025). <https://doi.org/10.1016/j.chempr.2025.102411>
[S19] M. Yang, J. Zhu, S. Bi, R. Wang, H. Wang et al., The construction of anion-induced solvation structures in low-concentration electrolyte for stable zinc anodes. Angew. Chem. Int. Ed. **63**(15), e202400337 (2024). <https://doi.org/10.1002/anie.202400337>
[S20] X. Liao, C. Pan, H. Yan, Y. Zhu, Y. Pan et al., Polyaniline-functionalized graphene composite cathode with enhanced Zn^2+^ storage performance for aqueous zinc-ion battery. Chem. Eng. J. **440**, 135930 (2022). <https://doi.org/10.1016/j.cej.2022.135930>
[S21] Z. Zhao, R. Wang, C. Peng, W. Chen, T. Wu et al., Horizontally arranged zinc platelet electrodeposits modulated by fluorinated covalent organic framework film for high-rate and durable aqueous zinc ion batteries. Nat. Commun. **12**, 6606 (2021). <https://doi.org/10.1038/s41467-021-26947-9>
[S22] Y. Quan, M. Yang, M. Chen, W. Zhou, X. Han et al., Electrolyte additive of sorbitol rendering aqueous zinc-ion batteries with dendrite-free behavior and good anti-freezing ability. Chem. Eng. J. **458**, 141392 (2023). <https://doi.org/10.1016/j.cej.2023.141392>
[S23] X. Zhao, N. Dong, M. Yan, H. Pan, Unraveling the interphasial chemistry for highly reversible aqueous Zn ion batteries. ACS Appl. Mater. Interfaces **15**(3), 4053–4060 (2023). <https://doi.org/10.1021/acsami.2c19022>
[S24] J.B. Park, C. Choi, J.H. Park, S. Yu, D.-W. Kim, Synergistic design of multifunctional interfacial Zn host toward practical Zn metal batteries. Adv. Energy Mater. **12**(48), 2202937 (2022). <https://doi.org/10.1002/aenm.202202937>
[S25] C. Xie, H. Ji, Q. Zhang, Z. Yang, C. Hu et al., High-index zinc facet exposure induced by preferentially orientated substrate for dendrite-free zinc anode. Adv. Energy Mater. **13**(3), 2203203 (2023). <https://doi.org/10.1002/aenm.202203203>
